# A Clinical Prediction Model for Pathologic Upgrade to Invasive Carcinoma Following Conization of Cervical High‐Grade Squamous Intraepithelial Lesions

**DOI:** 10.1002/cam4.70540

**Published:** 2024-12-30

**Authors:** Qiao Liu, Jing Yang, Hui Cheng, Chuqiang Shu, Yi Tang, Jing Zhao

**Affiliations:** ^1^ Hunan Provincial Maternal and Child Health Care Hospital University of South China Changsha Hunan People's Republic of China

**Keywords:** cervical cancer, HSIL, nomogram, pathological progression, predictive models

## Abstract

**Objective:**

To explore the risk factors associated with the pathological progression to invasive carcinoma following the conization of cervical high‐grade squamous intraepithelial lesions (HSIL) and to construct a risk prediction model to guide preoperative risk assessment and optimize the selection of surgical approaches.

**Methods:**

A retrospective analysis was conducted on the clinical data of 3337 patients who underwent cervical conization for HSIL at Hunan Provincial Maternal and Child Health Care Hospital from December 2016 to March 2022. The patients were categorized into the pathological progression group (398 cases) and the nonprogression group (2939 cases) based on postconization pathology results. Statistical significance factors were selected by least absolute shrinkage and selection operator regression and then multivariate logistic regression was utilized to build predictive models, which were presented as a nomogram and evaluated for discriminability, calibration, and decision curves. The Bootstrap method was utilized for internal validation. A total of 277 patients were enrolled from April 2022 to October 2022 for external validation.

**Results:**

The percentage of pathologic upgrades to invasive carcinoma following cervical conization was 11.9%. The predictive model included age, contact bleeding symptoms, HPV16/18 infection, HSIL cytology, cervical biopsy pathology diagnosis level, suspicious stromal infiltration in the biopsy pathology diagnosis, and endocervical curettage HSIL. The model demonstrated good overall discrimination in predicting the risk of HSIL progression to early invasive cancer, and internal validation confirmed its reliability (C‐index = 0.787). Area under the curve analysis indicated good model discriminability across external datasets. The decision curve analysis also suggested that this model is clinically useful.

**Conclusion:**

We developed and validated a nomogram incorporating multiple clinically relevant variables to better identify cases of HSIL progressing to early cervical cancer, providing a basis for individualized treatment and surgical approach selection.

## 1 Introduction

High‐grade squamous intraepithelial lesion (HSIL) of the cervix is a precancerous lesion, including cervical intraepithelial neoplasia (CIN) 2 and CIN 3, with CIN 3 having a higher risk of progression to cancer [1, 2, 3]. Aggressive treatment is clinically necessary. Cervical conization is the most common surgical approach. It not only directly removes lesions but also allows comprehensive reassessment of the extent of the patient's condition through pathological examination of the excised tissue, including the presence of invasive cancer. There is a trend toward younger patients suffering from cervical lesions, and the average age of women giving birth for the first time is increasing as a result of changes in the reproductive philosophy of women in the new era in China. Conization treatment increases the risk of future miscarriage and preterm birth, especially in those with midterm miscarriage [4, 5, 6]. Evaluating the immediate risk of cervical cancer prior to surgery can provide a basis for treatment decision‐making in order to protect female fertility. If the risk of invasive cervical cancer is low, delayed or ablative treatment may be considered. If the risk is high, conization surgery should be designed with sufficient scope to ensure an adequate negative margin, thereby avoiding the trauma of secondary surgery due to positive margins [7]. In addition, cervical conization is the preferred initial surgical approach for cervical HSIL; however, in clinical practice, informed selection of total hysterectomy is advisable. For example, conization is difficult in postmenopausal women with significantly atrophied cervix, as well as in cases where conization has been performed for HSIL and histology confirms HSIL lesion recurrence. Patients undergoing hysterectomy should be thoroughly assessed preoperatively to exclude the risk of cervical infiltration, avoiding insufficient surgical scope, which could complicate subsequent treatment or increase the risk of adverse outcomes. Through performing a retrospective analysis of clinical data from patients with cervical HSIL treated with conization, we aimed to explore the risk factors associated with post‐HSIL pathological progression to cervical invasive carcinoma, establish a risk prediction model, and assist clinicians in formulating treatment plans.

## 2 Materials and Methods

### 2.1 Data

A retrospective analysis was conducted on the clinical data of 3337 patients who underwent cervical conization for HSIL at Hunan Provincial Maternal and Child Health Care Hospital from December 2016 to March 2022. This included patients who underwent cervical biopsy for HSIL (including our hospital's colposcopy clinic biopsy or external hospital cervical biopsy reviewed by our pathology department). Among them, 398 had pathological progression to invasive cancer following cervical conization, whereas 2939 did not progress. Additionally, 277 HSIL cases were collected for external validation from April 2022 to October 2022 (12 cervical cancer and 265 HSIL following conization). The type of cervical conization was based on the type of transformation zone, defined as follows [8]: Type 1 excision resected type 1 transformation zone with a resection length of 7–10 mm; type 2 excision resected a type 2 transformation zone with a resection length of 10–15 mm; and type 3 excision resected a type 3 transformation zone with a resection length of 15–25 mm. Pathological progression was defined as follows: biopsy pathology result was HSIL, and postconization pathology result was cervical invasive cancer. Nonprogression was defined as follows: biopsy pathology result was HSIL, and postconization pathology result was HSIL or less (including cervical chronic inflammation, low‐grade squamous intraepithelial lesion, and HSIL). All the pathology slides were reviewed, and cases upgraded to invasive cancer were re‐staged according to the 2018 FIGO staging system for cervical cancer. HPV testing was conducted utilizing a nationally certified HPV detection method, categorized as HPV‐negative, high‐risk HPV‐positive (positive for HPV 16/18 types, high‐risk HPV infections other than HPV 16/18 types). Cytology reports were generated utilizing the TBS reporting system, including categories such as no intraepithelial lesion or malignancy (NILM), squamous epithelial cell abnormalities (ASC‐US, low‐grade squamous intraepithelial lesions; ASC‐H, HSIL), atypical glandular cells (AGC), and cervical cancer. Data collected included age, menopausal status, parity, clinical symptoms, HPV infection type, cytology, preoperative biopsy, and postconization pathology results. The inclusion criteria were biopsy pathology confirmed as cervical HSIL (CIN2‐3); preoperative gynecological ultrasound indicated no cervical mass; surgery method was cervical conization; postoperative pathology diagnosis was clear; and complete examination and treatment data were available. The exclusion criteria were incomplete data. There were no immunocompromised patients.

### 2.2 Statistical Analysis

Statistical analysis was performed utilizing R software (version 4.3.2; https://www.R‐project.org). The *χ* ^2^ test for the count data was conducted utilizing the R language package “CBCgrps.” The development of the nomogram model involved three steps. First, utilizing nonzero coefficients in the least absolute shrinkage and selection operator (LASSO) regression model, independent predictive features were identified [9]. Second, the variables selected in the LASSO regression model were identified, a multivariate logistic regression model was constructed, and statistically significant variables were selected for nomogram modeling [10]. Third, the discrimination and calibration of the nomograms were assessed utilizing calibration graphs, area under the receiver (AUC), and Harrell's consistency index (C‐index). The nomogram underwent bootstrapping validation (1000 bootstrap resamples) to calculate a relatively corrected C‐index [11].

### 2.3 Ethical Approval

This study was approved by the Human Ethics Committee of Hunan Provincial Maternal and Child Health Care Hospital (ethics approval number K2022034) and was performed in accordance with the tenets of the Declaration of Helsinki. The requirement for written informed consent was waived by the Human Ethics Committee of Hunan Provincial Maternal and Child Health Care Hospital because the data were anonymized and retrospectively analyzed.

## 3 Results

### 3.1 Baseline Characteristics and Univariate Analysis

**FIGURE 1:**
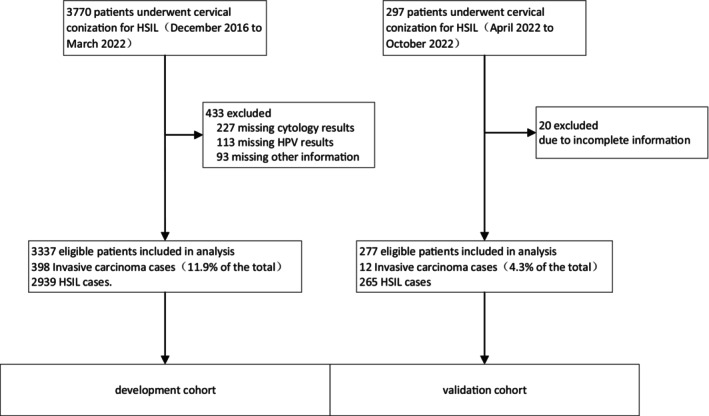
Flowchart for the development and validation of predictive model.

A flowchart of the patient selection and assignment process is presented in Figure 1.

Of the 3337 patients with cervical HSIL, 398 cases (11.9%) experienced pathological progression to cancer following conization. According to the 2018 FIGO staging for cervical cancer, there were 335 cases of cervical squamous cell carcinoma (SCC) IA1, 39 cases of SCC IA2, 11 cases of SCC IB1, two cases of cervical adenosquamous carcinoma (ASC) IA1, five cases of cervical adenocarcinoma (AC) IA1, two cases of cervical AC IA2, and four cases of cervical AC IB1 in the progression group. Stratified analysis by age showed that the proportion of patients aged ≥ 50 years in the progression group was 38%, which was significantly higher than that of 26% in the nonprogression group. The univariate analysis revealed significant differences between the two groups in terms of age, menopausal status, parity, contact bleeding symptoms, HPV16/18 infection, HSIL cytology, cervical biopsy pathology grade, involvement of glandular tissue, suspicious stromal infiltration in biopsy pathology diagnosis, and ECC HSIL (*p* < 0.05; Table 1).

**TABLE 1:** Results of univariate analysis of various clinical and pathologic parameters of patients in both groups.

Variables	Invasive carcinoma (*n* = 398)	HSIL (*n* = 2939)	Total (*n* = 3337)	*p*
Age (years)				
< 30	17 (4)	242 (8)	259 (8)	< 0.001
30–39	95 (24)	1007 (34)	1102 (33)	
40–49	135 (34)	913 (31)	1048 (31)	
≥ 50	151 (38)	777 (26)	928 (28)	
Menopause				
No	281 (71)	2377 (81)	2658 (80)	< 0.001
Yes	117 (29)	562 (19)	679 (20)	
Parity (times)				
< 2	169 (42)	1494 (51)	1663 (50)	0.002
≥ 2	229 (58)	1445 (49)	1674 (50)	
Gravidity (times)				
< 3	102 (26)	956 (33)	1058 (32)	0.007
≥ 3	296 (74)	1983 (67)	2279 (68)	
Colporrhagia				
No	307 (77)	2525 (86)	2832 (85)	< 0.001
Yes	91 (23)	414 (14)	505 (15)	
Vaginal_fluid				
No	392 (98)	2899 (99)	3291 (99)	0.995
Yes	6 (2)	40 (1)	46 (1)	
HPV type				
Type 16 and/or 18	200 (50)	942 (32)	2195 (66)	< 0.001
Other HR‐HPV types or negative	198 (50)	1997 (68)	1142 (34)	
Preoperative cytology				
Non‐HSIL	131 (33)	1876 (64)	2007 (60)	< 0.001
HSIL	267 (67)	1063 (36)	1330 (40)	
Cervical biopsy				
CIN2	45 (11)	1060 (36)	1105 (33)	< 0.001
CIN2‐3	160 (40)	1159 (39)	1319 (40)	
CIN3	193 (48)	720 (24)	913 (27)	
Gland involvement				
No	230 (58)	1913 (65)	2143 (64)	0.005
Yes	168 (42)	1026 (35)	1194 (36)	
Suspicious mesenchymal infiltration				
No	319 (80)	2784 (95)	3103 (93)	< 0.001
Yes	79 (20)	155 (5)	234 (7)	
Endocervical curettage				
Non‐HSIL	169 (42)	2263 (77)	2432 (73)	< 0.001
HSIL	229 (58)	676 (23)	905 (27)	

*Note:* Cytology in the HSIL group includes ASC‐H, HSIL, AGC, and cervical cancer without a clear diagnosis.

### 3.2 Feature Selection and Multivariate Analysis

**FIGURE 2:**
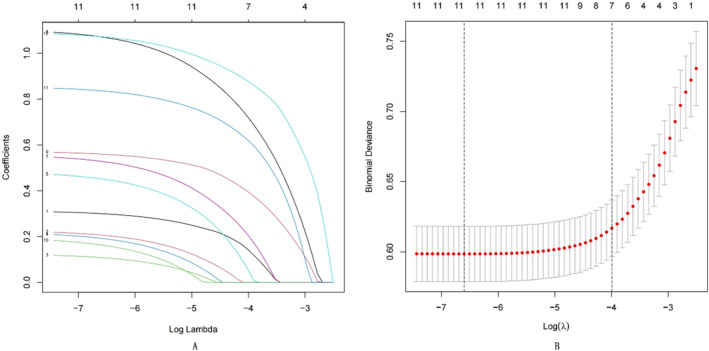
(A) The feature selection was used for LASSO. LASSO coefficient profiles of the 12 features. Coefficient profiles were plotted based on the log(λ) series. (B) The *x*‐axis represents the logarithmic value of lambda, the *y*‐axis represents mean square error (MSE), and the values above the graph represent the number of independent variables. The two vertical dashed lines represent the logarithmic values of lambda corresponding to the minimum mean square error and the lambda value corresponding to one standard error away from the minimum mean square error. The optimal λ produced 11 features with non‐zero coefficients.

LASSO regression analysis imposes constraints on model parameters by shrinking the regression coefficients of certain variables to zero, thereby minimizing the prediction error of quantitative response variables. The LASSO method excluded variables with zero regression coefficients from the model, retaining those with the strongest correlation with the response variable. This method selected 11 optimal predictive factors for the regression model, which were consistent with those identified during univariate screening. Subsequently, a multivariate logistic regression model was constructed utilizing these 11 variables (Figure 2).

Multivariate regression analysis showed that the index, including age, contact bleeding symptoms, HPV 16/18 infection, HSIL cytology, cervical biopsy pathology grade, suspicious stromal infiltration in biopsy pathology diagnosis, and ECC HSIL, were independent risk factors for invasive carcinoma following HSIL conization of the uterine cervix (*p* < 0.05; Table 2). Ultimately, the prediction model was built utilizing seven variables with *p* values < 0.05 in the multivariate regression analysis.

**TABLE 2:** Multivariate logistic analysis.

	*β* coefficient	Odds ratio (95% CI)	*p*
Age	0.314	1.369 (1.155–1.626)	0.000
Colporrhagia (yes)	0.486	1.626 (1.214–2.162)	0.001
HPV type (16/18)	0.560	1.751 (1.385–2.214)	0.000
Preoperative cytology (HSIL)	1.108	3.028 (2.39–3.853)	0.000
Cervical biopsy	0.574	1.775 (1.499–2.106)	0.000
Suspicious mesenchymal infiltration (yes)	0.857	2.356 (1.674–3.296)	0.000
Endocervical curettage (HISL)	1.094	2.987 (2.361–3.781)	0.000

### 3.3 Nomogram and Evaluation of the Prediction Model for Pathological Progression to Invasive Carcinoma Following Conization

**FIGURE 3:**
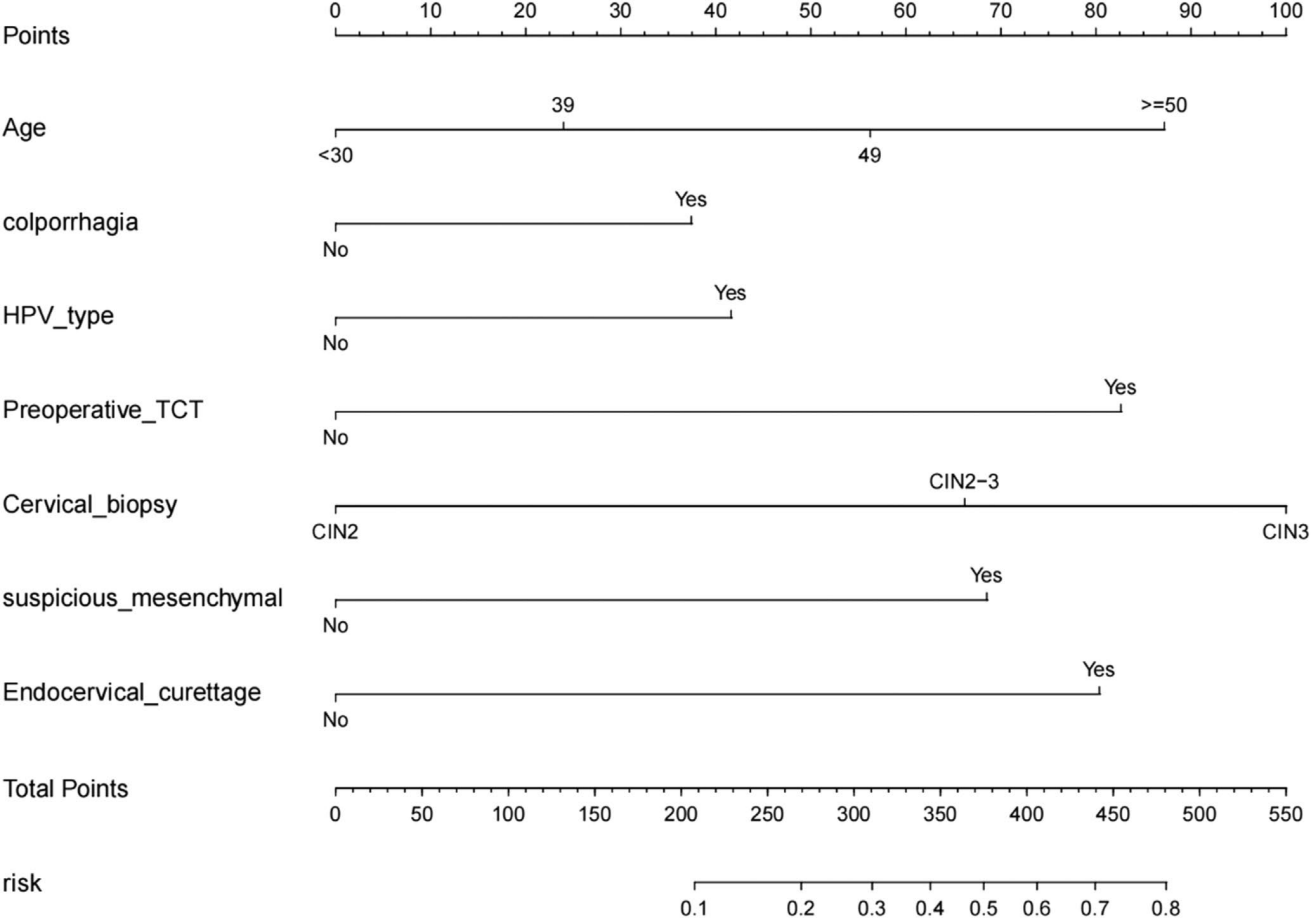
The nomogram of invasive carcinoma following HSIL conization of the cervix. The risk of predicting the occurrence of invasive carcinoma is quantified as the number of points marked on the axis, the score determined by each variable axis is the number corresponding to the value vertical on the total points scale, and projected the sum of all variables onto the bottom axis, yielding a personalized invasive carcinoma risk for each woman with HISL.

The scores corresponding to every predictive factor are shown in the column model (Figure 3), with every variable quantified by drawing a vertical line on the point axis. The total score of the model is the sum of individual scores.

**FIGURE 4:**
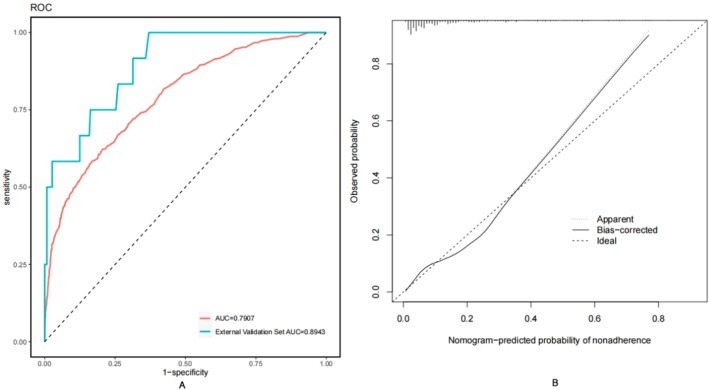
(A) The ROC curves showing the precision of the invasive cancer nomogram in patients. The AUC of the nomogram was 0.7907, when the risk probability is 0.149 as the cutoff point, the validation model's sensitivity and specificity were 0.623 and 0.794, respectively. The AUC for the external validation set is 0.8943. (B) The *y*‐axis represents the actual diagnosed invasive cancer. *x*‐axis represents the predicted risk of invasive cancer. The diagonal dashed line represents the perfect prediction of the ideal model. A closer fit of the prediction curve to the diagonal dashed line indicates a well‐calibrated model.

Figure 4 illustrates the predictive performance of the proposed model. The AUC of the receiver operating characteristic (ROC) curve was 0.790, with a threshold of 0.149 (0.623, 0.794), where the sensitivity and specificity of the model were maximized at 62.3% and 79.4%, respectively. The AUC for the external validation set was 0.8943, indicating that the model demonstrated good discriminability. The Hosmer–Lemeshow goodness‐of‐fit test was utilized to assess the calibration of the predictive model, and the calibration curve demonstrated good consistency in predicting the risk of invasive cancer following HSIL conization. The C‐index of the prediction nomogram was 0.793, which was confirmed as 0.787 through internal validation (Bootstrapping), indicating the good discrimination ability of the model.

### 3.4 Decision Curve Analysis and Clinical Use

**FIGURE 5:**
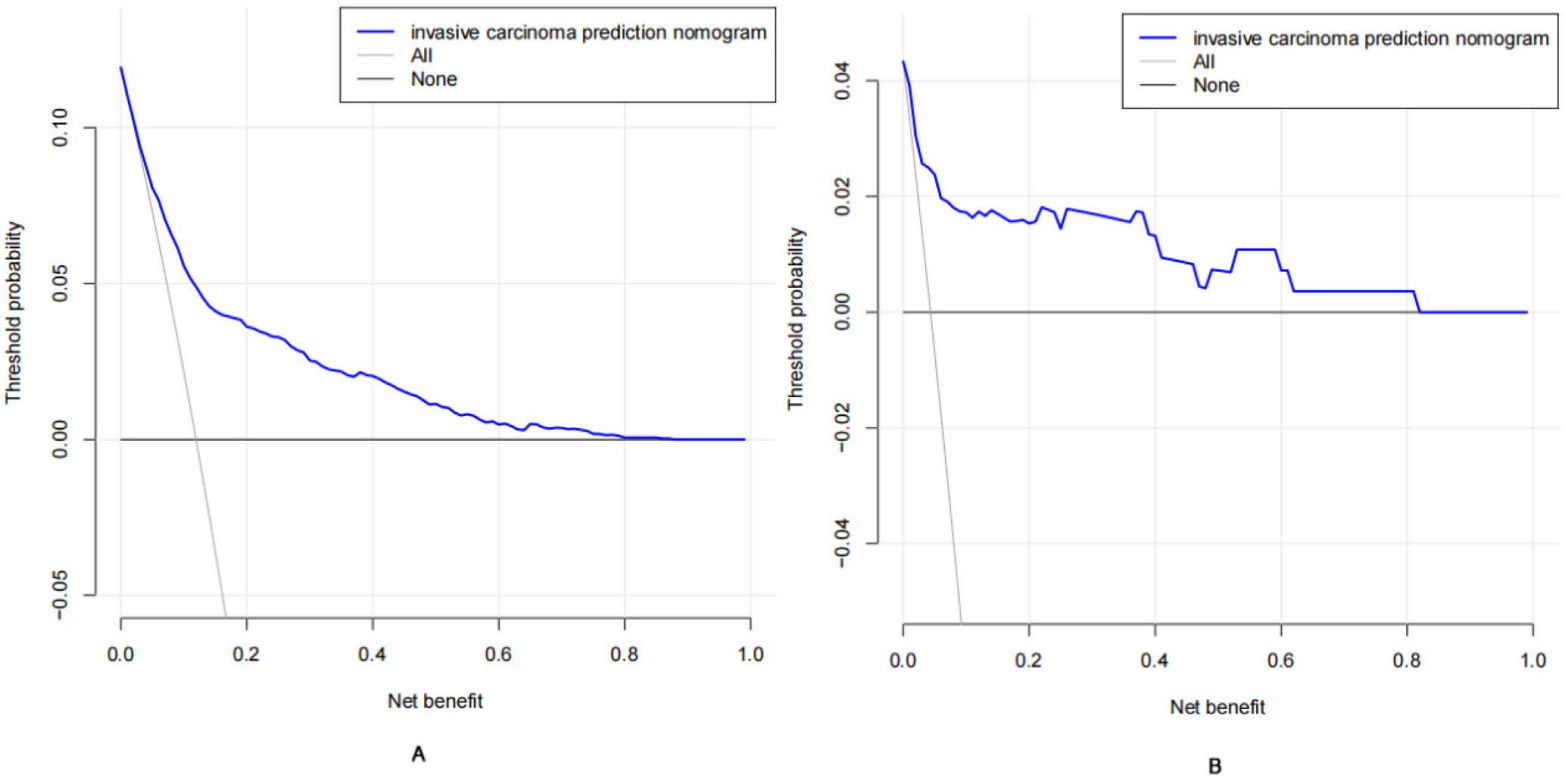
Decision curve analysis (DCA) for invasive cancer nomogram. (A) The DCA curve is in the transverse range from 0.01 to 0.87, the DCA curve lies above the None and All null lines, indicating that the model is moderately effective in this range; in the range of less than 0.1 or greater than 0.87, the DCA curve is close to the None and All null lines, indicating that the model is less effective in this range. (B) The external validation set.

The DCA of the nomogram for invasive cancer is presented in Figure 5. The DCA curve was in the lateral range of 0.1–0.87, indicating that the model was moderately effective in this range. In the range of < 0.1 or > 0.87, the DCA curve is close to the zero line of None and All, thus indicating that the model performs poorly in this range. The external validation set indicates that the developed model is potentially clinically useful.

## 4 Discussion

Cervical HSIL is associated with a risk of progression to cancer [3]. Currently, it is challenging to predict whether HSIL will progress to invasive lesions, and some patients may already have occult cervical cancer. For young women, there is a need to balance the potential benefits of treatment with future pregnancy risks, aiming to slow or reduce the scope of excisional surgery for the purpose of achieving pregnancy in the future. In older individuals who are not candidates for cervical cone biopsy or who undergo other hysterectomy‐related procedures [12], assessing the risk of cervical cancer is crucial to avoid underdiagnosis of cervical cancer, leading to a reduction in the scope of surgery. Therefore, we conducted a retrospective analysis to identify factors contributing to the upgrade of cervical HSIL to invasive cancer and developed a novel predictive nomogram for preoperative assessment, providing a basis for personalized treatment and surgical method selection.

We utilized LASSO regression analysis to screen predictive factors, followed by multifactor logistic regression analysis. Independent risk factors for cervical cone biopsy upgrade to invasive cancer were identified, including age, symptoms of contact bleeding, HPV16/18 infection, HSIL cytology, pathological grade of HSIL in cervical biopsy, pathological diagnosis indicating suspicious stromal infiltration, and ECC HSIL. The contribution of every predictive factor to the outcome event (size of regression coefficients) was utilized to assign corresponding scores, which were cumulated to obtain the total score. The risk of developing invasive cancer was then calculated based on the relationship between the total score and the probability of the outcome event. Nomogram models can be utilized to predict the risk of disease and prognosis. Because of their perfect representation of the weight of every factor through the length of the lines, these models do not require complex function transformations during use, thereby providing convenience in clinical applications. For example, a 40‐year‐old woman with contact bleeding, positive for HPV16/18 and ASC‐H on cytology, presents with a vaginal colposcopy impression of HSIL (SCJ fully visible) and a cervical biopsy revealing CIN2, with negative ECC results. Gynecological ultrasound or pelvic magnetic resonance imaging findings indicate no cervical mass. The patient has urgent fertility plans. According to the risk assessment based on the nomogram models, the risk of concurrent cervical invasive cancer is 10%. After informing the patient of the risks, options such as cervical ablation surgery or postponement of surgery (with biannual follow‐up) can be considered. For example, a patient with a biopsy‐confirmed diagnosis of HSIL who has a high risk of invasive carcinoma according to nomogram models needs to have as much cervical tissue removed as possible when performing cervical conization to avoid positive margins. Older patients with a biopsy‐confirmed diagnosis of HSIL tend to want a total hysterectomy. According to the risk assessment based on the nomogram model, the risk of combined invasive carcinoma is very low, and such patients may be able to dispense with a single cervical conization to rule out invasive carcinoma and undergo a total hysterectomy. Nomogram models have been less frequently applied to patients with cervical cancer, where they have been primarily utilized to predict lymph node metastasis and cervical cancer prognosis [13, 14]. To the best of our knowledge, no previous reports have described nomogram models for predicting cervical cone biopsy‐upgraded cervical HSIL to invasive cancer. Our study focused on patients diagnosed with cervical HSIL via biopsy, exploring factors contributing to the upgrade of cervical HSIL to cervical invasive cancer, and constructing a nomogram model. Following comprehensive assessment and internal validation, the model demonstrated good discriminative ability, calibration, and high clinical utility.

Cervical HSIL may coexist with cervical cancer, and the risk of combined cancer increases with age [15]. Wetrich [16] discovered that the detection rates of cervical cancer in HSIL diagnosed via cervical biopsy for patients aged 15–20, 21–25, 26–30, 31–40, and 41–50 years were 3%, 8%, 13%, 23%, and 25%, respectively. In our study, patients aged < 30, 30–39, 40–49, and 50 years diagnosed with HSIL via cervical biopsy had detection rates of 4.3%, 23.9%, 33.9%, and 37.9%, respectively. The result was statistically significant, consistent with prior research. Prior studies [17, 18] have suggested that with increasing age, new squamocolumnar junctions (SCJs) move into the cervical canal, reducing the visibility of the SCJ during colposcopy. When high‐grade lesions coexist with cancer, cervical cancer often appears at the top edge of HSIL [19]. In 41.6%–57.4% of cervical cancers, the SCJ is not visible or the lesion extends into the cervical canal [20, 21]. We reached the same conclusion that ECC HSIL is an independent risk factor for cervical HSIL progression to cancer. Persistent infection by high‐risk HPV is a key factor in the development of cervical tumors. Gu et al. [22] found that HPV16/18 infection exhibited stronger persistence, attributing 69.1% of cervical invasive cancers to HPV16/18 infection [23]. Our study revealed a greater likelihood of upgrading cervical HSIL to cancer in patients with HPV16/18 infection. The 2014 “TBS Report on Cervical Cytology” [24] suggested that as the severity of cervical cytological examination results increases, the risk of developing invasive cancer also increases. The clinical risks of ASC‐H and AGC are similar to those of HSIL [25] and are uniformly classified as high‐grade lesions. The 2019 ASCCP Consensus Guidelines stated that patients with cytology results indicating high‐grade lesions have a risk of being diagnosed with HSIL or HSIL+ > 25% [26]. In our study, the rate of HSIL cytology in the upgraded group was significantly higher than that in the cervical HSIL group, confirming its role as an independent risk factor for pathological progression to cancer, consistent with prior research [27]. Cervical HSIL and early‐stage cervical cancer often have insufficient symptoms, with contact bleeding being a common manifestation. Detecting lesions relies on routine cervical cancer screening. Studies suggest that 0.7%–39% of women with cervical cancer experience postcoital bleeding, and evidence from a series of cases indicates that women with postcoital bleeding are more likely to have cervical cancer than the general population [28], consistent with our study. Therefore, when patients present with clinical symptoms, vigilance is required to detect early invasive cancer in conjunction with cervical HSIL. The 2014 WHO Classification of Female Reproductive Organs Tumors (4th edition) combined CIN 2 and 3 into the histological diagnosis of HSIL [29], with CIN 3 having a higher risk of progression to cancer [1, 2, 3]. Our study also found that the risk of upgrading to invasive cancer differed when the cervical biopsy results indicated CIN2, CIN2–3, and CIN3. Specifically, the risk of upgrading to invasive cancer following cone biopsy was 2.4 times higher for CIN2–3 and 3.7 times higher for CIN3 compared with CIN2. Local stromal infiltration extended several millimeters deep, with no fixation [30] or specific features [31]. Therefore, during colposcopy, early cervical cancer detection in large‐area HSIL lesions via biopsy is a random event. When pathological diagnosis suggests suspicious stromal infiltration, extra caution is necessary for the development of invasive cancer.

Numerous studies have suggested a positive correlation between the size of the lesion area and the severity of the lesion [16, 32, 33, 34, 35, 36, 37, 38]. Unfortunately, the colposcopic results were not included in the study because they came from different medical institutions and were too subjective to standardize the evaluation criteria. Consequently, the calculation of the colposcopic lesion area could not be performed, and the quadrant of lesion involvement was not included in the study because of the large differences in the level of the colposcopies. Therefore, it is necessary to gradually include more variables to improve the model and to increase the amount of multicenter data to better serve the clinic.

## 5 Conclusion

In this study, we preliminarily constructed and validated a predictive model for the pathological upgrading of cervical HSIL to invasive cancer following conization by incorporating multiple clinically relevant variables. This approach may help clinicians provide personalized treatment for patients with cervical HSIL. However, this study is retrospective, with limitations, such as unsatisfactory data and different colposcopy results from different medical institutions. These shortcomings may affect the accuracy of the results and require further comprehensive prospective multicenter clinical studies.

## Author Contributions

**Qiao Liu:** conceptualization (equal), data curation (equal), formal analysis (equal), investigation (equal), methodology (equal), validation (equal), writing – original draft (equal), writing – review and editing (equal). **Jing Yang:** data curation (equal), methodology (equal), validation (equal), writing – original draft (equal). **Hui Cheng:** data curation (equal), investigation (equal), validation (equal), writing – original draft (equal). **Chuqiang Shu:** project administration (equal), resources (equal), writing – review and editing (equal). **Yi Tang:** data curation (equal), formal analysis (equal), methodology (equal), writing – review and editing (equal). **Jing Zhao:** conceptualization (equal), formal analysis (equal), investigation (equal), methodology (equal), project administration (equal), writing – original draft (equal), writing – review and editing (equal).

## Conflicts of Interest

The authors declare no conflicts of interest.

## Data Availability

The data that support the findings of this study are available on request from the corresponding author. The data are not publicly available due to privacy or ethical restrictions.
